# The LKB1 Tumor Suppressor Controls Spindle Orientation and Localization of Activated AMPK in Mitotic Epithelial Cells

**DOI:** 10.1371/journal.pone.0041118

**Published:** 2012-07-18

**Authors:** Chongjuan Wei, Varun Kumar Bhattaram, John C. Igwe, Elizabeth Fleming, Jennifer S. Tirnauer

**Affiliations:** 1 Department of Epidemiology, The University of Texas MD Anderson Cancer Center, Houston, Texas, United States of America; 2 Center for Molecular Medicine, University of Connecticut Health Center, Farmington, Connecticut, United States of America; 3 Neag Comprehensive Cancer Center, University of Connecticut Health Center, Farmington, Connecticut, United States of America; Northwestern University Feinberg School of Medicine, United States of America

## Abstract

Orientation of mitotic spindles plays an integral role in determining the relative positions of daughter cells in a tissue. LKB1 is a tumor suppressor that controls cell polarity, metabolism, and microtubule stability. Here, we show that germline LKB1 mutation in mice impairs spindle orientation in cells of the upper gastrointestinal tract and causes dramatic mislocalization of the LKB1 substrate AMPK in mitotic cells. RNAi of LKB1 causes spindle misorientation in three-dimensional MDCK cell cysts. Maintaining proper spindle orientation, possibly mediated by effects on the downstream kinase AMPK, could be an important tumor suppressor function of LKB1.

## Introduction

Orientation of the mitotic spindle within a dividing cell is a highly regulated process that impacts both cell and tissue architecture [1], [2], [3]. A core group of conserved cell polarity mediators related to the *c elegans* partitioning defective (PAR) proteins control the spindle orientation process [3], [4], [5], [6]. PAR homologs recruit a Leucine-Glycine_asparagiNe rich protein (LGN) to specific regions of the cell cortex in a cell-type-dependent manner to determine whether the spindle will align along the apico-basal or the planar cell axis [7]. These and additional cortical factors then facilitate the capture of astral microtubules emanating from the spindle poles, which in turn leads to the production of force for spindle translocation and rotation. Planar spindle orientation results in side-by-side placement of daughter cells, a characteristic pattern in epithelial cell sheets.

In addition to the core polarity mediators, several tumor suppressor proteins involved in cytoskeletal and cell polarity regulation have also been shown to play roles in planar spindle orientation. These include the Adenomatous polyposis coli (APC), Von Hippel Lindau (VHL), and E-cadherin tumor suppressors [8], [9], [10], [11]. The mechanisms by which these proteins orient spindles and their relationship to the core spindle orientation machinery are incompletely understood. All of these proteins interact with microtubules as well as components of the actin cortex and associated polarity proteins, and they could thus regulate spindle orientation by a number of mechanisms.

A mammalian homolog of *c elegans* Par4 is the Liver Kinase B1 (LKB1), encoded by the *STK11* gene. This gene acts as a tumor suppressor in humans. Germline mutations in *STK11* cause Peutz Jeghers Syndrome (PJS), an autosomal dominant disorder characterized by gastrointestinal hamartomatous polyps and a dramatically increased risk for the development of a variety of cancers [12], [13], [14], [15]. *STK11* mutations and LKB1 loss of function are also found in many other sporadic cancers including lung and cervical cancer [14], [16], [17], [18], [19], [20], [21], [22].

The LKB1 protein is a serine-threonine kinase that phosphorylates members of the AMP activated kinase (AMPK) related family, which in turn link energy metabolism to cell polarization [15], [23], [24]. These LKB1 substrates include AMPK, microtubule affinity regulating (MARK) kinases, and synapse of the amphid defective (SAD) kinases, among others [15], [24], [25]. Phosphorylation of these substrates by LKB1 increases their activity. Which of these substrates is responsible for LKB1 tumor suppressor function has not been determined unequivocally, although the role of AMPK function in cancer has recently received attention [26], [27], [28], [29].

Apart from its role in cancer, LKB1 and homologues in lower organisms contribute to the establishment of cell polarity [30], [31]. In worms, Par4 mutants show dramatic alterations in morphogenesis throughout embryonic development including the first embryonic cell division [32],[33]. The fly homolog dLKB1 controls oocyte polarity and embryonic axis specification [34]. *STK11* gene knockout in mice causes polarity defects in several tissues, in addition to producing tumors in the gastrointestinal tract and mammary gland [35], [36], [37], [38], [39]. In single epithelial cells in culture, activation of LKB1 through its association with the pseudokinase STRAD was able to induce autonomous cell polarization with asymmetric distribution of cortical polarity markers; LKB1 is the only protein for which this activity has been demonstrated [40]. LKB1 was recently shown to affect cortical actin in a kinase-independent manner through activation of RhoA [41]. These effects on cell polarity could affect spindle orientation by altering important cortical interaction sites for astral microtubules.

Spindle orientation also depends on astral microtubule elongation to the cell cortex and dynamics, and LKB1 effects on microtubule function have recently been appreciated. Loss of LKB1 increases microtubule polymerization from centrosomes [42],[43], suggesting the major effect of LKB1 is to destabilize microtubules. LKB1 can destabilize microtubules by phosphorylating AMPK or MARKs, which in turn promote dissociation of Tau and Clip170 and microtubule associated proteins (MAPs) 2 and 4 from the microtubule lattice [44], [45], [46], [47], [48]. These effects on microtubules appear to have functional consequences, despite the fact that loss of LKB1 function does not produce dramatic changes in the appearance microtubule array [42], [49]. LKB1 regulation of astral microtubule elongation and dynamicity could also contribute to spindle orientation.

Finally, LKB1 has been noted to regulate mitotic spindle structure. Loss of LKB1 function induced multipolar spindle formation and aneuploidy in hematopoietic stem cells and fly neuroblasts, and reduction of spindle microtubule density and asymmetric astral microtubule distribution in neuroblasts [50], [51]. Interestingly, LKB1 did not play a role in spindle rotations in the fly neuroblast system [32].

These roles for LKB1 in cell polarity, microtubule stability, and mitosis suggested that loss of LKB1 function might cause spindle misorientation in tumors with *STK11* mutation. We tested this by analyzing spindles in tissues from *STK11* mutant mice and in MDCK cells grown three-dimensionally in Matrigel. We found that reduced LKB1 function caused spindle misorientation in both systems. This misorientation could be reproduced in MDCK cysts by treating cells with Compound C, a relatively nonspecific inhibitor of AMPK [52]. In vivo, *STK11* mutation caused the activated form of AMPK to be mislocalized in dividing cells, from mitotic spindle poles in wild-type tissues to the cell cortex in *STK11* mutant tumors. These findings suggest that LKB1 plays an important role in orienting spindles in epithelial cells, and that controlling the activation or localization of AMPK might be a primary mechanism by which LKB1 regulates spindle orientation.

## Results

To determine the role of LKB1 in mitotic spindle orientation in vivo, we assayed mitotic spindle angles in mice carrying a germline inactivating *STK11* mutation [53]. These mice are heterozygous for the mutation (homozygous deletion is lethal during embryogenesis) and phenocopy most features of human Peutz Jeghers syndrome including spontaneous intestinal tumor formation [12], [17], [35], [54], [55]. We collected small tumors (polyps) from *STK11* mutant mice aged 9–11 months and corresponding gastric/duodenal tissues from wild-type littermates, fixed and embedded them in OCT, and cut sections that were thick enough to encompass entire mitotic spindles within the section. We used immunofluorescence for microtubules and staining for DNA to image spindles, and stained cortical actin to mark the apical brush border as an orientation landmark. We measured spindle angle relative to the apical brush border, defining an angle of 0° as exactly parallel to this surface. We limited the analysis to metaphase and anaphase spindles, for which orientation is fully established in wild-type animals [8], [56].

**Figure 1:**
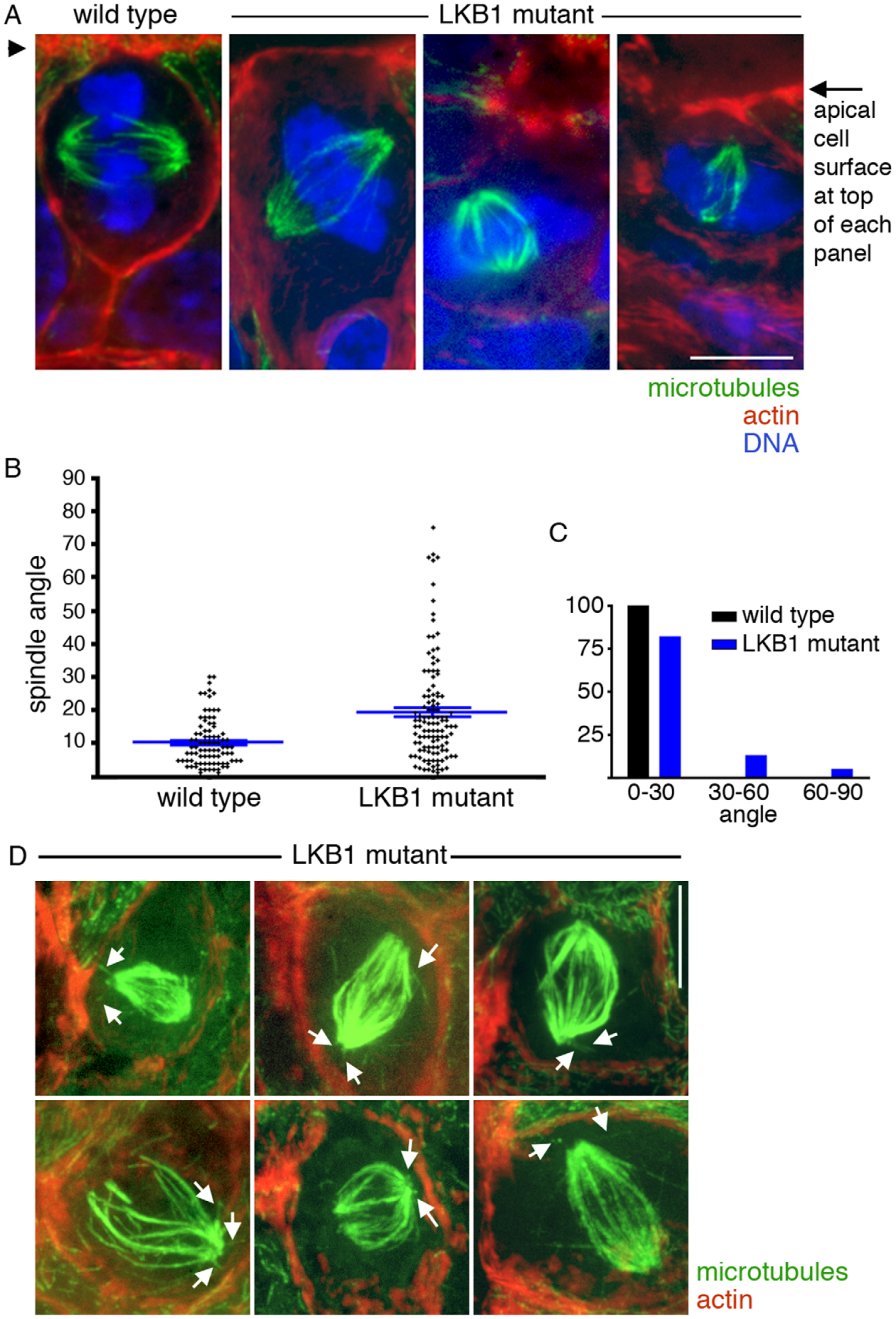
*STK11* mutation causes spindle misorientation in vivo. Tissue immunofluorescence was done on upper gastrointestinal tumors from *STK11*/LKB1 heterozygous mutant mice and corresponding gastrointestinal tissue from wild-type littermates. A) Representative images from *STK11*/LKB1 wild-type tissues and *STK11*/LKB1 mutant polyps. The brush border at the apical cell surface is at the top of each panel (arrowhead at left and arrow at right) and the spindles were rotated in three dimensions to place both spindle poles in a single plane. Microtubules are green, actin is red, and DNA is blue. Spindle angle was measured as the angle between the spindle axis and the apical surface. The wild-type spindle is oriented parallel to the apical surface, and the three *STK11*/LKB1 mutant spindles lack this planar orientation. B) Quantification of spindle angles. Each dot represents a single spindle angle measurement. Blue bars represent means and standard error of the mean. See text for numbers. P<0.0001 for the difference. C) Data from (B) presented as the % of angles that range from 0° to 30°, 30° to 60°, and 60° to 90°. D) Spindles from *STK11* mutant cells contain astral microtubules. White arrows show astral microtubules of misoriented spindles in *STK11*/LKB1 mutant polyps. These spindles are from the original data set used to calculate spindle angle, but the confocal stacks were adjusted, rotated, and processed differently from (A) to show astral microtubules optimally, and spindle angle cannot be appreciated from them. Scale bars, 10 µm.

Analysis of spindles from wild-type upper gastrointestinal tissues showed planar spindle orientation, as we had found previously in the lower gastrointestinal tract [8], [56]. The mean spindle angle in controls was 10°±7° (n = 90 spindles from 5 animals), and the highest angle was 30°, seen in only two cells (Figure 1). In contrast, spindles in cells from *STK11* mutant tumors showed a large range of spindle angles. The mean spindle angle was 19°±16° (n = 117 spindles from 7 animals), a highly statistically significant difference from controls (P = 7×10^−8^). Spindle angles greater than 30° were seen in all tumors (Figure 1). These spindle angle measurements demonstrate that *STK11* mutation leads to spindle misorientation in vivo.

Tumors evolve over several weeks to months in LKB1 mutant animals, during which time additional genetic changes could occur that could be responsible for spindle abnormalities. Thus, we assessed whether a short term LKB1 RNAi approach would also produce spindle misorientation. We first analyzed the effect of LKB1 RNAi on monolayers of MDCK cells, a cell line with well-established spindle orientation regulation, grown on Transwell filters [5], [57], [58], [59], [60]. This showed a barely appreciable spindle orientation defect (mean angle of 4° in controls versus 7° upon LKB1 RNAi, p = 0.02; data not shown).

Despite the fact that MDCK cells grown on Transwell filters polarize well, growth on filters generates a two-dimensional epithelium, and three-dimensional growth can reveal polarity regulation not seen in two-dimensions [58]. We therefore tested spindle orientation in the MDCK cyst system, in which single cells are plated on a bed of Matrigel and allowed to form hollow cysts. These cystic structures are spherical monolayers of polarized cells with the apical cell surfaces facing inward towards a single hollow lumen and the basal cell surfaces facing outward to contact the Matrigel, and they more closely mimic the geometry of the epithelial structures that form in vivo [58].

**Figure 2:**
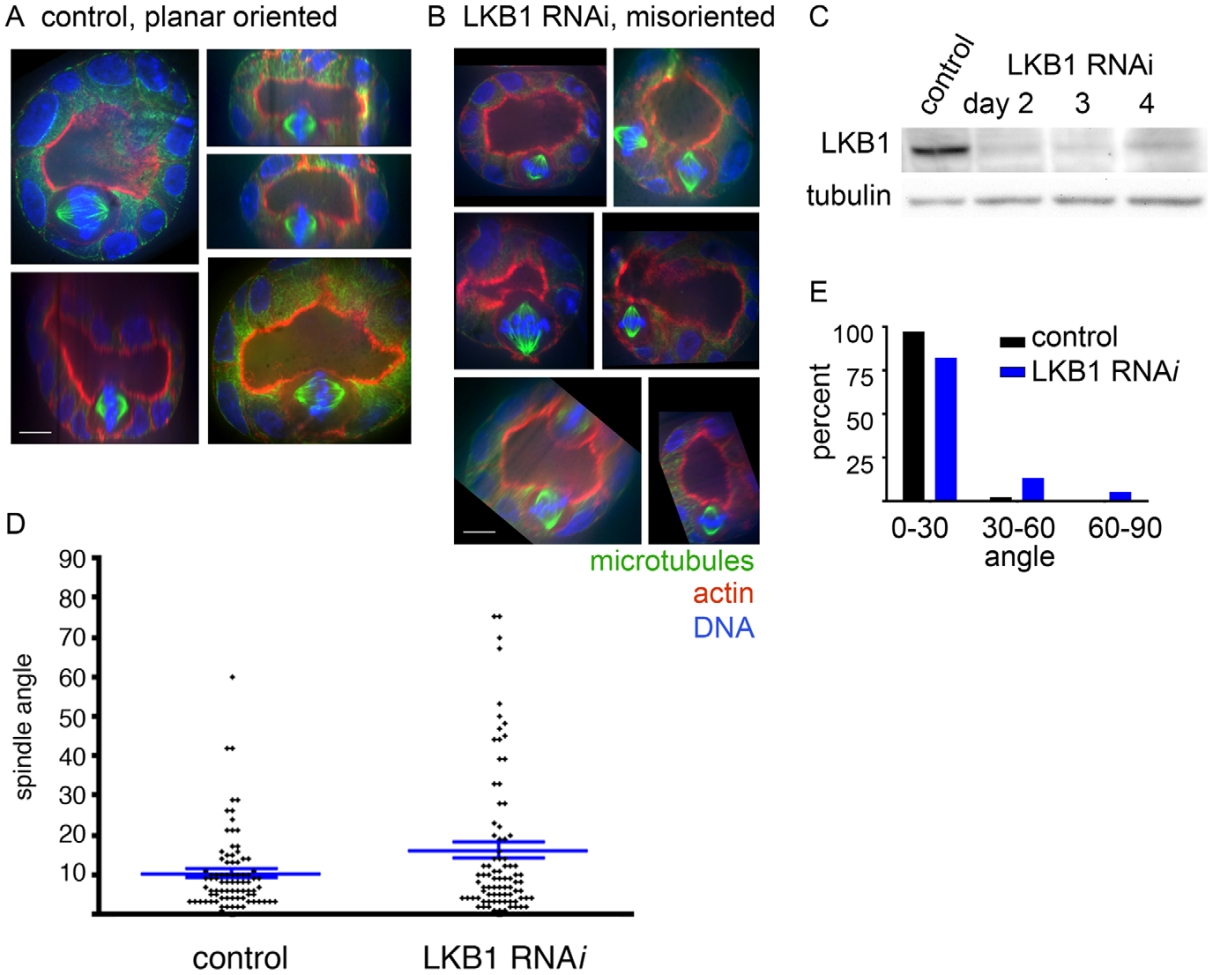
LKB1 RNAi causes spindle misorientation in MDCK cell cysts. A) Representative spindles from control RNAi cysts. Images were rotated in three dimensions to include the cyst lumen, place the apical cell surface at the top of the panel, and display both spindle poles in a single plane. Spindle angle was measured relative to the apical surface. Microtubules are green, actin is red, and DNA is blue. B) Representative spindles from LKB1 RNAi cysts, displayed as in (A). Scale bar, 10 µm. C) Western blot showing RNAi samples from cells lysed at the time of cyst fixation. Tubulin is blotted as a loading control. D) Quantification of spindle angles. Each dot represents a single spindle angle measurement. Blue bars represent means and standard error of the mean. See text for numbers. P<0.0001 for the difference. E) Data from (D) presented as the % of angles that range from 0° to 30°, 30° to 60°, and 60° to 90°.

In three-dimensional cyst culture, LKB1 RNAi caused a pronounced spindle orientation defect similar to that seen in vivo. Control cysts showed planar spindle orientation, with a mean spindle angle of 10±10° (n = 86 spindles from 3 experiments), while cysts with LKB1 RNAi showed spindle misorientation, with a mean spindle angle of 16±18° (18% of spindle angles were >30°, n = 84 spindles from 3 experiments; Figure 2). Of note, this is a similar magnitude of spindle orientation defect as we saw with RNAi of the tumor suppressor adenomatous polyposis coli (APC) in this same MDCK cyst system (mean angle 18±17°, with 35% of spindles >30°; data not shown). This data supports the conclusion that LKB1 function directly contributes to planar spindle orientation without intervening genetic changes.

**Figure 3:**
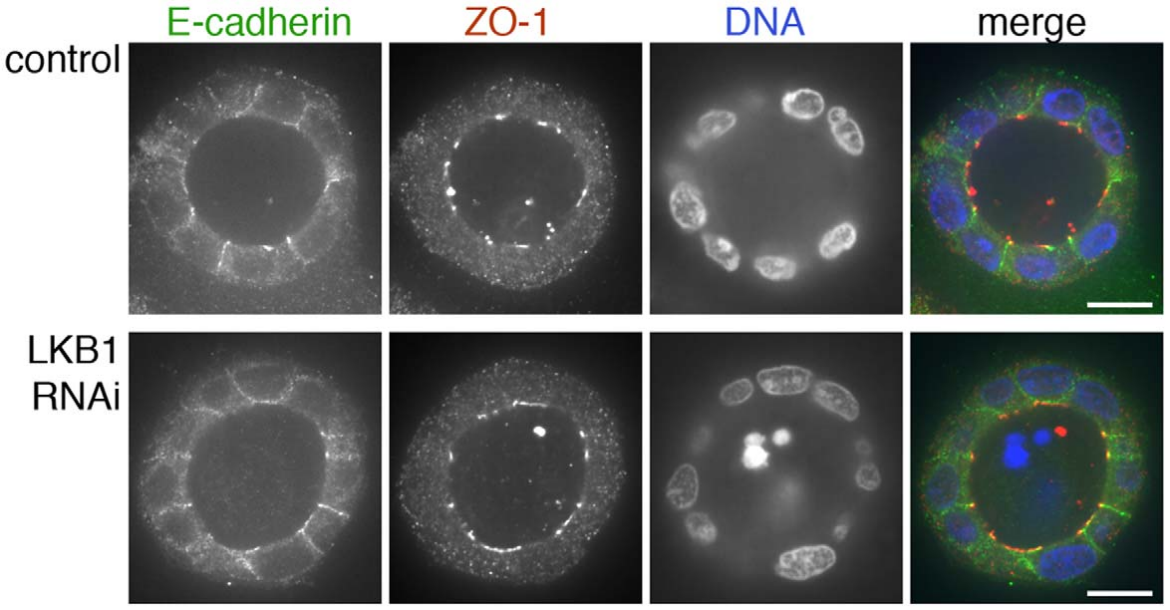
ZO-1 and E-cadherin localization are unaffected by LKB1 RNAi in MDCK cell cysts. Representative images of ZO-1 (red) and E-cadherin (green) immunofluorescence in control MDCK cell cysts and in cysts with LKB1 RNAi are shown. Sections were taken through the midpoint of the cyst to show the hollow lumen as well as the apical and lateral surfaces of cells at the widest part of the cyst structure. Bars, 10 µm.

To further investigate the mechanism by which LKB1 controls spindle orientation, we assayed for changes in cell polarity markers. In tumors from *STK11* mutant mice, cortical actin, ZO-1, and β-catenin localization appeared normal (Figure 1 and data not shown). Analysis of control and LKB1 RNAi cysts likewise showed that the localization and appearance of cortical actin, as well as the tight junction component ZO-1 and the adherens junction mediator E-cadherin were unaffected by LKB1 reduction. This included the formation of an actin brush border at the cells’ apical/luminal surface, localization of ZO-1 at apical borders and apical cell-cell boundaries, and localization E-cadherin along lateral cell surfaces (Figures 2 and 3). Thus, LKB1 control of spindle orientation appears to be mediated through factors that function downstream of cell-cell junctions and the associated cell polarity machinery rather than by controlling cell-cell junction formation or actin cortex organization.

We previously found that APC mutation caused spindle misorientation without eliminating astral microtubules, suggesting that loss of other components of the spindle orientation machinery besides astral microtubules was responsible for the misorientation [8]. Although we were not able to do an extensive quantitative analysis, we also found that astral microtubules were present in cells from *STK11* mutant animals (Figure 1D). This suggests that the role of LKB1 in spindle orientation is in control of the orientation machinery rather than in elongation of astral microtubules, although it does not rule out effects of LKB1 on astral microtubule dynamics.

Since cortical markers and astral microtubule length appeared unaffected by LKB1 loss of function, we next looked at LKB1 substrates to identify potential mediators of the role of LKB1 in spindle orientation. We focused on AMPK as one of the best characterized LKB1 substrates; AMPK also has a potential cancer association [22], [29]. We assayed the localization of activated (phosphorylated) AMPK, which has been shown to localize to spindle poles in cultured cell lines and tumors [61], [62].

**Figure 4:**
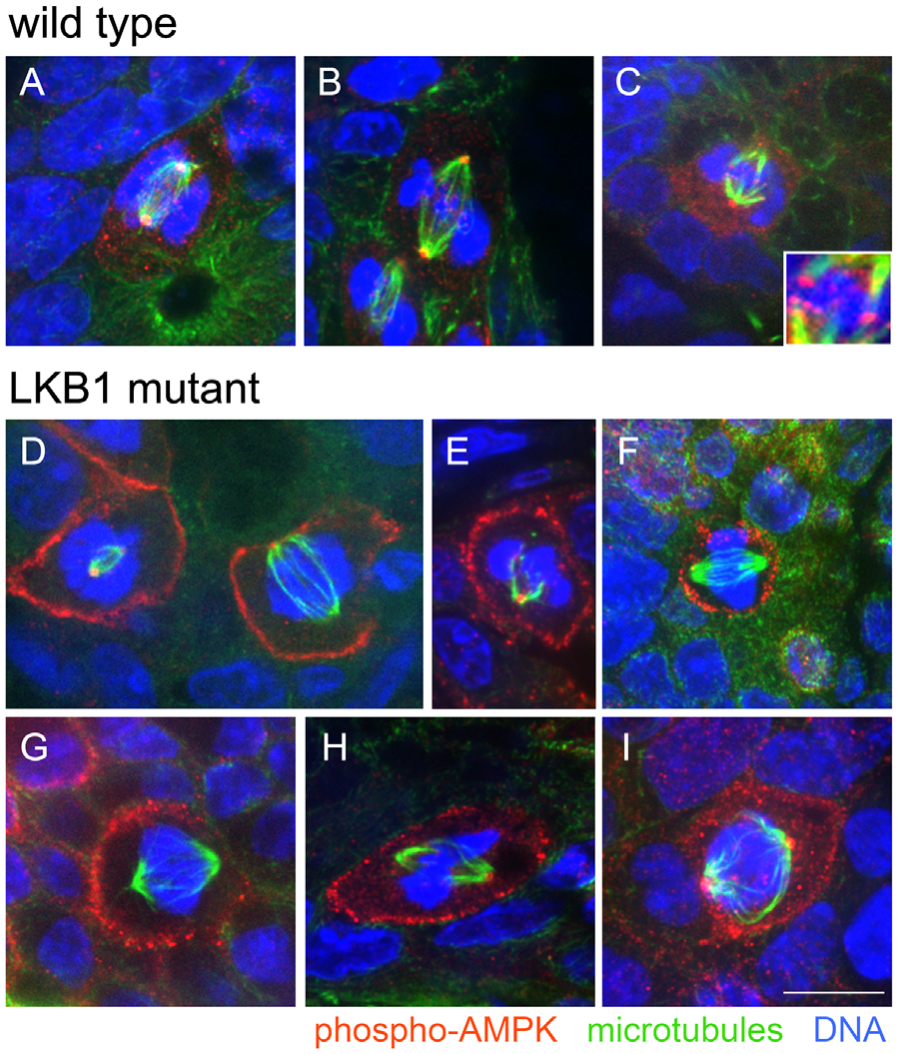
LKB1 mutant tumors show mislocalization of activated AMPK. A–C) Representative images of phospho-Thr172-AMPK (red) localization in mitotic cells from wild-type tissues. Images were not rotated, and not all cells had an obvious apical surface in the tissue section. The inset in C shows the apparent localization of phospho-AMPK to kinetochore regions. Microtubules are green and DNA is blue. D–I) Representative images of phospho-Thr172-AMPK (red) localization in mitotic cells from LKB1 mutant polyps. Images were not rotated, and not all cells had an obvious apical surface in the tissue section. Microtubules are green and DNA is blue. Scale bar, 10 µm.

In cells from wild-type animals, an antibody to AMPK phosphorylated at Thr-172 (the site of phosphorylation by LKB1) showed the expected localization to mitotic spindle poles (Figure 4). In contrast, this antibody showed a new and dramatically different localization in LKB1 mutant tissues. A majority of mitotic cells from LKB1 mutant animals showed localization of phospho-AMPK to the cell cortex, either combined with spindle pole localization or alone (Figure 4). For wild-type tissues, 75% of mitotic cells showed localization of phospho-AMPK to the spindle poles, and 6% showed localization to the cell cortex; these localizations were not mutually exclusive (n = 134 mitotic cells from 4 animals). The finding of rare cortical localization in controls suggests the possibility that the cell cortex is a physiological site for phospho-AMPK localization that is transient.

In *STK11* mutant tissues, 70% of mitotic cells showed localization of phospho-AMPK to spindle poles and 68% showed cortical localization (n = 130 cells from 5 animals). Thus, *STK11* mutation caused dramatic mislocalization of activated AMPK in addition to spindle misorientation. Of note, we also saw apparent kinetochore localization of phospho-AMPK in 10–15% of mitotic cells from both wild type and *STK11* mutant animals (Figure 4C, inset). Thus, LKB1 mutation produced cortical localization of phospho-AMPK without perturbing other sites of activated AMPK localization in mitotic cells.

While *STK11* mutation caused mislocalization of activated AMPK in tissues, we did not see this mislocalization in MDCK cell cysts with LKB1 RNAi. Further, depolymerization of astral microtubules with low-dose Nocodazole in MDCK cyst cells did not reproduce this mislocalization (data not shown). Thus, additional features of the tumorigenic process must be involved in this activated AMPK mislocalization.

To test for a role of AMPK in controlling spindle orientation, we attempted to reduce AMPK level or function in MDCK cysts by a variety of specific methods. However, for various technical reasons, these were unsuccessful (see Methods). Thus, we used the pharmacologic agent Compound C, which is often used as a specific AMPK function-blocking tool, but which in fact inhibits several other important signaling pathways [52], [63]. Since AMPK is reported to play a role in tight junction formation in MDCK cells [64], [65], we applied Compound C to pre-formed MDCK cell cysts in which tight junctions have already been formed.

**Figure 5:**
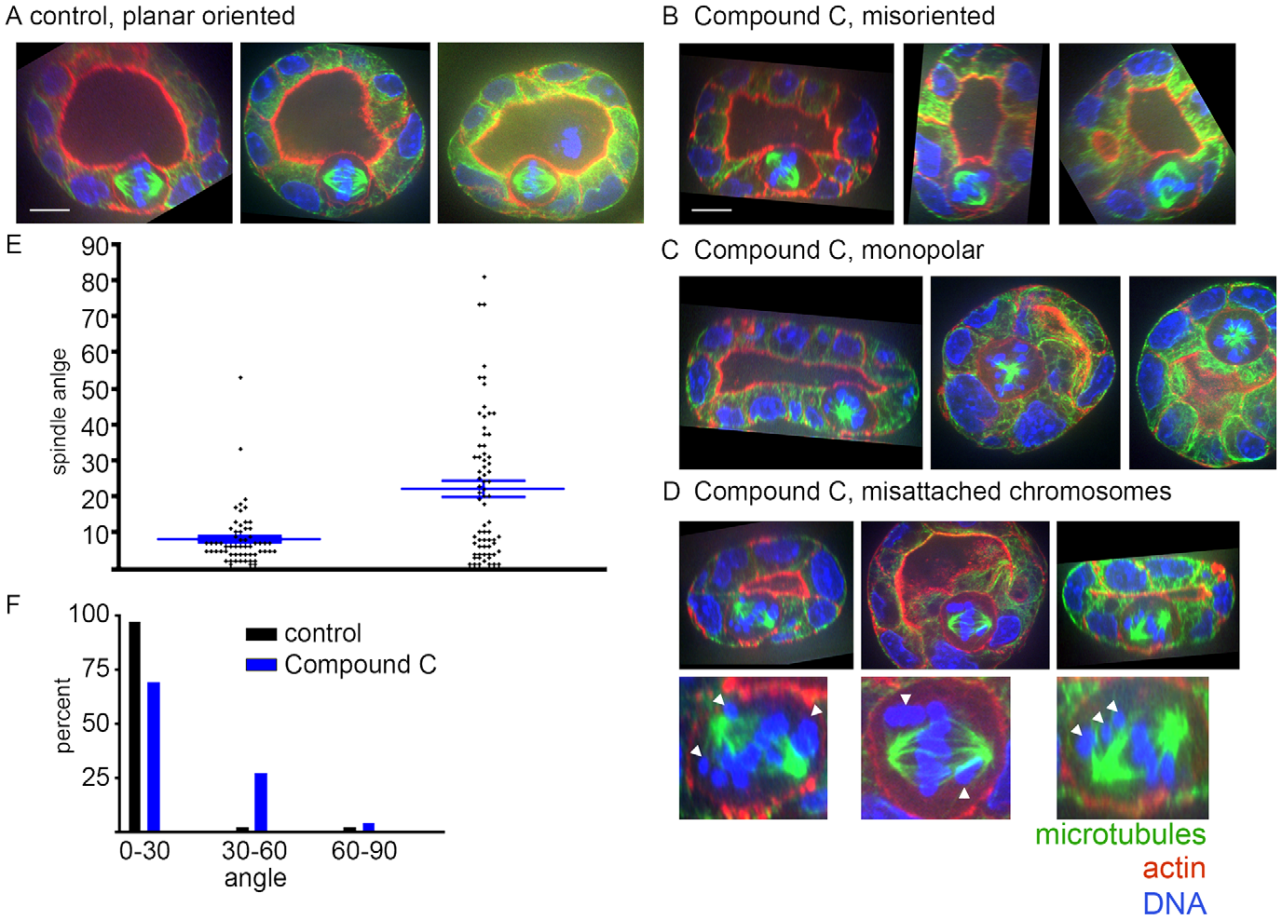
AMPK inhibition by Compound C causes spindle misorientation and other mitotic defects in MDCK cell cysts. A) Representative spindles from vehicle treated control cysts. Images were rotated in three dimensions to show the cyst lumen, place the apical cell surface at the top of the cell, and place both spindle poles in a single plane. Spindle angle was measured relative to the apical surface. Microtubules are green, actin is red, and DNA is blue. B–D) Representative spindles from cysts treated with 20 µM Compound C for 4 hours prior to fixation. Images in B are displayed as in A. Images in C and D are taken from single lumen cysts, although the lumen is not visible in every image. Insets in D show magnified cells with misattached chromosomes marked by white arrows. Scale bar, 10 µm. E) Quantification of spindle angles. Each dot represents a single spindle angle measurement. Blue bars represent means and standard error of the mean. See text for numbers. P<0.0001 for the difference. F) Data from (E) presented as the % of angles that range from 0° to 30°, 30° to 60°, and 60° to 90°.

Compound C treatment for 3.5–4 hours resulted in significant spindle misorientation, with a mean spindle angle in controls of 8±8° (n = 62 spindles from 3 experiments) and a mean angle of 22±19° in Compound C treated cyst cells (n = 70 spindles from 3 experiments; Figure 5). This demonstrates a similar degree of spindle misorientation upon AMPK inhibition as with LKB1 RNAi. Of note, Compound C treatment also caused an increased incidence of monopolar spindles (27% of spindles (22 of 83); Figure 5C) and misattached chromosomes (28% of spindles (17 of 61); Figure 5D), which did not occur with LKB1 RNAi. These findings suggest that AMPK could be a mediator of the role of LKB1 in spindle orientation and could also have additional mitotic roles.

## Discussion

### A Role for LKB1 in Planar Spindle Orientation

Our analysis of gastrointestinal tissues in vivo and three-dimensional MDCK cysts grown in Matrigel establishes a role for LKB1 in planar spindle orientation in physiologically polarized epithelial cell monolayers.

Our analysis of upper gastrointestinal cells from wild-type animals showed robust planar (aligned along the plane of the tissue axis) spindle orientation, as we found previously in small intestinal epithelium [8], [56]. We note that this is differs from the findings of other groups, who observed either planar orientation in transit amplifying cells (TACs) and apico-basal orientation in intestinal stem cells, or complete lack of spindle orientation regulation in intestinal epithelium [9], [66]. An explanation for these differing results has not yet become apparent. Technical differences in tissue processing or spindle and cell cortex imaging might explain some of the discrepancies. Alternatively, real and informative biological differences might exist between the animals used by different groups.

LKB1 mutation in tissues and RNAi in three-dimensional cell culture both produced numerous misoriented spindles, although neither of these manipulations completely abolished spindle orientation regulation. The degree of spindle misorientation with LKB1 loss of function might appear small when comparing the mean spindle angle deviation from control, but the range of spindle angles produced by LKB1 loss of function was large and consistent across experiments. This is also consistent with other studies of spindle orientation, in which manipulations of spindle orientation proteins rarely randomize spindle angle, and typically produce abnormalities of this magnitude [8], [10], [67]. It is likely that redundancies among spindle orientation mediators and the contribution of cell geometry partially rescue the phenotype of loss of any individual component involved in the process.

### Contribution of AMPK to Spindle Orientation

Our data is consistent with a mechanism of spindle orientation by LKB1 that involves the downstream kinase AMPK. LKB1 could regulate AMPK function by promoting its activation or its targeting to specific cellular locations. AMPK loss of function in tumors could reduce apico-basal cell polarity, astral microtubule attachments to the cell cortex, or both of these. Our data did not show significant effects of LKB1 loss of function on cell polarity. Thus, we favor a model in which LKB1 affects astral microtubule interactions or force generation at the cell cortex through AMPK. However, further studies are needed to test this.

In vivo, it appears that the presence of phospho-AMPK at the cell cortex is associated with spindle misorientation. However, LKB1 RNAi in MDCK cell cysts did not cause mislocalization of activated AMPK, suggesting that the mislocalization of AMPK is not required for spindle misorientation. In contrast, blocking AMPK function by Compound C treatment in cysts caused spindle misorientation, suggesting a possible role for AMPK function in the spindle orientation process. If AMPK is indeed the true downstream mediator of LKB1 role in spindle orientation, there are several possible effectors, including microtubule binding proteins such as CLIP170 and Tau. Roles for other AMPK related family members such as MARKs are not excluded by our data. Further studies are needed to test these mechanisms.

We did not determine how activated AMPK becomes mislocalized in LKB1 mutant tissues. Based on the occasional finding of phospho-AMPK at the cell cortex in normal tissues from wild-type animals, we favor the idea that this is a physiologic localization that occurs transiently or at a low level compared to spindle pole localization, and that this localization is enhanced upon loss of LKB1 function. We did not see this mislocalization of activated AMPK in APC mutant tumors (data not shown), suggesting it is specific for LKB1 loss of function.

We also do not know how AMPK activation occurs in cells with LKB1 mutation. AMPK might be activated by residual LKB1 due to retained heterozygosity for the wild-type *STK11* gene; by other known AMPK activators such as calcium/calmodulin dependent protein kinase kinase (CAMKK) or TGF-β activating kinase 1 (TAK1); by as yet unidentified AMPK activators; or by inhibition of AMPK phosphatases [63], [68]. Further studies will be needed to determine what mediates localization of AMPK to spindle poles and what activates AMPK in the absence of LKB1.

Despite the difference between activated AMPK localization in tumors and MDCK cysts, we demonstrated that loss of AMPK function caused spindle misorientation, using Compound C treatment. Compound C also caused additional mitotic defects not seen with LKB1 mutation in tissues or LKB1 RNAi in cysts. Potential explanations for monopolar spindles and misattached chromosomes by Compound C include a more complete inhibition of AMPK by the drug than of LKB1 function by heterozygous mutation and RNAi, roles for AMPK that are compensated upon LKB1 mutation, or roles for other kinases that are inhibited by Compound C. Interestingly, chromosome defects were seen upon loss of LKB1 and AMPK function in other cell types, so it will be important to determine the relationship between LKB1 and AMPK in other mitotic processes [50].

### Connection between Spindle Misorientation and Cancer

LKB1 joins several important tumor suppressors (APC, VHL, E-Cadherin) in regulating the spindle orientation process. Spindle orientation by LKB1 and these other tumor suppressors could converge on a common pathway that influences the core spindle orientation machinery, or each tumor suppressor could act independently. Arguing against a shared mechanism between LKB1 and APC is the observation that the APC binding partner β-catenin was mislocalized in APC mutant tumors, while it was localized normally in LKB1 mutant tumors ([8], [35] and our data not shown). Conversely, phospho-AMPK was mislocalized in LKB1 mutant tumors, but localized normally to mitotic spindle poles and not the cell cortex in tumors from APC^min^ mice (data not shown). However, neither of these findings rule out a convergence of LKB1 and APC function on a further downstream factor or on proteins that link astral microtubules to the correct cortical sites. Understanding the hierarchy and interactions among tumor suppressors that regulate spindle orientation is an important goal for the future.

While evidence from mouse models of polycystic kidney disease demonstrate that spindle misorientation alone does not initiate tumor formation, spindle misorientation has the potential to impact several major aspects of tumor biology including tissue hypertrophy, metastasis, and the size of the cancer stem cell compartment [1]. Our study does not address the role of LKB1 in spindle orientation in intestinal stem cells versus differentiated cells, but recent report showed that LKB1 regulates stem cell self-renewal and prevents stem cell exhaustion in the hematopoietic lineage [50]. It will be interesting to determine whether LKB1 plays a similar role in epithelial stem cell biology, and whether spindle misorientation contributes to tumor formation in *STK11* mutant epithelial tissues.

## Materials and Methods

### Ethics Statement

This study was approved by the Animal Care Committee (ACC) at the University of Connecticut Health Center and by the Institutional Animal Care and Use Committee (IACUC) at the MD Anderson Cancer Center.

### *STK11* Mutant Mice and Tissue Immunofluorescence

*STK11* heterozygous mutant mice were generated as previously described in the C57BL/6J strain background [53]. Mice were genotyped by tail vein DNA, and tissues from *STK11* heterozygotes and wild-type littermates were harvested at 9–11 weeks as described in [8]. Interestingly, the remaining *STK11* allele sometimes remains wild-type in these tumors [35], [69]. Intestinal tissues were fixed as described in [56]. Briefly, the entire excised gastrointestinal tract was washed with PBS, fixed in 4% formaldehyde freshly made from paraformaldehyde (Ted Pella, Redding, CA), infused with 30% sucrose (Fisher Scientific, Pittsburgh, PA), and embedded in OCT (Triangle Biomedical Sciences, Durham, NC). Polyps were microdissected and embedded separately. Tissue histology was confirmed by pathologic review of hematoxylin and eosin-stained sections. Frozen sections (15 µm) of gastric tissue and individual polyps were cut onto tissue path slides (Fisher Scientific).

Immunofluorescence was performed using directly labeled antibodies to α-tubulin (FITC-DM1A, Sigma Aldrich, St. Louis, MO). DNA was stained with DRAQ5 (Biostatus, Liecestershire, UK) and Hoechst 33342 (Sigma Aldrich). Actin was stained with Alexa^568^-labeled phalloidin (Invitrogen, Eugene, OR). Activated AMPK was detected using an antibody to AMPK phosphorylated at Thr-172 (40H9, Cell Signaling Technology, Danvers, MA; this antibody recognizes a single band of appropriate size in Western blots), with Alexa^568^-conjugated anti-rabbit secondary antibody (Invitrogen/Molecular Probes). Coverslips were mounted using the Prolong Gold reagent (Invitrogen/Molecular Probes, Carlsbad, CA).

### MDCK Cell Culture and Cyst Formation

An MDCK cell clone capable of forming three-dimensional cysts was a generous gift of Ian Macara (University of VA; derivation of these cells has been described in previously published papers [70], [71]). Cells were grown in MEM with 5% FCS. Cyst growth was initiated by plating 5,000 well-trypsinized cells on a bed of growth factor reduced Matrigel (BD Biosciences, San Jose, California) in home-made chambers made from segments cut from a 25 ml pipette that were adhered to glass slides with vacuum grease. Cells were overlaid with 2% Matrigel in growth media. Cysts formed from single cells over the course of 4 days. For all spindle orientation experiments, the proteasome inhibitor MG132 (EMD/Calbiochem) was added to a final concentration of 20 µM to accumulate cells in metaphase 3 hours prior to fixation or at the time of drug addition.

### LKB1 RNAi

MDCK cells were transfected with an siRNA directed against the canine *STK11* 5′ untranslated region (sequence GGGCAGCAGATAAGGGAGA) or control siRNA purchased from Dharmacon (Lafayette, CO). Transfection was done by electroporation with an Amaxa Nucleofector (Lonza, Williamsport, PA) in solution T with waveform T23 according to the manufacturer’s instructions. Cells from the same transfection were used for three-dimensional cyst plating and Western blotting. Prior to cyst plating cells were allowed to recover in normal growth media for 6–8 hours.

### AMPK Loss of Function

Our analysis showed that the MDCK cells used in this study express the AMPKα1 isoform and not AMPKα2 (data not shown). Thus, we attempted AMPK loss of function experiments in MDCK cell cysts using several specific reagents that targeted AMPKα1, including siRNAs, multiple rounds of infection with lentiviral shRNA, transfection of locked nucleic acid (LNA)-based siRNA, and transfection of a dominant negative AMPKα2 mutant [72]. Because the cells used to make cysts are multiply antibiotic resistant, none of these constructs could be selected for or stably transfected. None of these approaches was able to reduce AMPK level or function to the degree or duration needed to impair AMPK function in the MDCK cyst assay (data not shown).

For Compound C treatment, Compound C (EMD/Calbiochem, Gibbstown, NJ) at 20 µM or an equal volume of the diluent DMSO was added to pre-formed cysts at day 4 of growth, 3.5–4 hours prior to fixation.

### MDCK Cyst Immunofluorescence

Cysts were fixed in 4% formaldehyde for one hour. Immunofluorescence for α-tubulin and phospho-Thr-AMPK, and staining of actin and DNA, was done as described for tissue sections. For E-cadherin and ZO-1 immunofluorescence, cysts were fixed in methanol for 30 minutes, and immunofluorescence done with antibodies to E-cadherin (13-900, Invitrogen) and ZO-1 (40-2300, Zymed Laboratories, San Francisco, CA) incubated overnight, followed by Alexa fluor-conjugated secondary antibodies, and DNA staining as above.

### Western Blotting

Cells for Western blotting were grown in standard tissue culture dishes and harvested at the time of cyst fixation by trypsinization, washing with PBS, pelleting, and freezing at −80°C. Lysates were made by thawing cells in lysis buffer (50 mM Hepes pH 7.4, 150 mM NaCl, 1 mM MgCl_2_, 1 mM EGTA, 1% Triton-X100 and protease inhibitors Leupeptin, Pepstatin, Chymostatin, NaV0_4_, NaF, and PMSF) for 30 minutes and collecting the supernatent following centrifugation at 16,000 x G for 30 minutes at 4°C. SDS-PAGE was done on 10% acrylamide gels loaded with 50 µg of protein per sample with transfer to PVDF membranes (Millipore, Billerica, MA). Western blotting was done with antibodies to LKB1 (27D10, Cell Signaling Technology) and α-tubulin (DM1A, Sigma) and developed with Supersignal Fempto reagent (Pierce, Rockford, IL).

### Confocal Imaging and Spindle Angle Analysis

Imaging of tissue sections and MDCK cell cysts prepared as above was done on an inverted Nikon TE2000-U microscope (Nikon Instruments; Melville, NY) equipped with a Yokogawa CSU-10 spinning disk confocal head (Perkin Elmer; Wellesley, MA) and a deep-cooled Orca AG CCD camera (Hamamatsu Photonics; Bridgewater, NJ), using a 1.4 NA Plan Apo 100x objective. Z-sections were acquired at 0.2 µm intervals using an electronic stepping motor (Prior Scientific Inc.; Rockland, MA). Image acquisition, processing, and analysis were controlled by MetaMorph software (Molecular Devices Corp.; Sunnyvale, CA).

For tissue sections, we measured spindle angles in all sequential mitotic cells in metaphase and anaphase in wild-type mouse intestinal tissues and polyps from *STK11* mutant mice for which an apical cell surface could be clearly defined. For MDCK cell cysts we only analyzed spindles in cysts with a single lumen, as judged by Phalloidin staining, and a single layer cell thickness; these made up the majority of cysts. These criteria probably slightly underestimate the magnitude of spindle misorientation by discounting cysts that have the most severely altered morphology as a consequence of spindle misorientation. We chose to use these criteria to eliminate any spindles misoriented due to abnormal cyst morphology rather than to the experimental manipulation and to eliminate possible mis-calculation of spindle angle in cysts with multiple lumens.

Spindle angle was measured as the angle between the spindle axis and the brush border along the cell’s apical surface, using the MetaMorph region measurements tool. For every cell, an image that contained both of the spindle poles and the maximum amount of apical cell surface in a single plane was generated using the MetaMorph three-dimensional reconstruction feature (for tissues) or orthogonal stack/orthogonal planes feature (for MDCK cell cysts). An angle of 0° was defined as a spindle axis parallel to the apical surface, and 90° as a spindle axis perpendicular to the apical surface. All angles were recorded as positive numbers.

We analyzed 90 spindles from 5 control animals and 117 spindles from 7 *STK11* mutant animals. For MDCK cell cyst experiments, we analyzed 80–90 spindles from three independent experiments for each manipulation. Mean spindle angles with standard deviation as well as spindle angle range were determined for each condition. Statistical significance was determined by two-sided T test.

All images shown in the Figures are unmanipulated except for rotating panels to provide a uniform viewing convention and increasing brightness and contrast to improve visibility while preserving linearity of the signal (without adjustment of the gamma parameter) – except for the images of astral microtubules (Figure 1D). For these images of astral microtubules only, the gamma parameter was adjusted to show the presence of astral microtubules without over-saturating the signal from the spindle microtubules.
